# Performance of Dysmorphology‐Based Screening for Genetic Disorders in Pediatric Congenital Heart Disease Supports Wider Genetic Testing

**DOI:** 10.1002/mgg3.70040

**Published:** 2024-11-25

**Authors:** Benjamin M. Helm, Lindsey R. Helvaty, Erin Conboy, Gabrielle C. Geddes, Brett H. Graham, Melissa Lah, Leah Wetherill, Benjamin J. Landis, Stephanie M. Ware

**Affiliations:** ^1^ Department of Medical and Molecular Genetics Indiana University School of Medicine Indianapolis Indiana USA; ^2^ Department of Epidemiology Indiana University Richard M. Fairbanks School of Public Health Indianapolis Indiana USA; ^3^ Department of Pediatrics Indiana University School of Medicine Indianapolis Indiana USA

**Keywords:** congenital heart disease, dysmorphology, screening performance

## Abstract

**Background:**

Dysmorphology evaluation is important for congenital heart disease (CHD) assessment, but there are no prior investigations quantifying the screening performance compared to standardized genetics evaluations. We investigated this through systematic dysmorphology assessment in CHD patients with standardized genetic testing in primarily pediatric patients with CHD.

**Methods:**

Dysmorphology evaluations preceding genetic testing results allowed us to test for associations between dysmorphic status and genetic diagnoses while adjusting for extracardiac anomalies (ECAs). We use a test‐negative case–control design on a pediatric inpatient CHD cohort for our study.

**Results:**

Of 568 patients, nearly 96% of patients completed genetic testing, primarily chromosome microarray (CMA) ± exome sequencing‐based genetic testing (493/568, 86.8%). Overall, 115 patients (20.2%) were found to have genetic diagnoses, and dysmorphic patients had doubled risk of genetic diagnoses, after ECA adjustment (OR = 2.10, *p* = 0.0030). We found that 7.9% (14/178) of ECA−/nondysmorphic patients had genetic diagnoses, which increased to 13.5% (26/192) in the ECA−/dysmorphic patients. Nearly 43% of ECA+/dysmorphic patients had genetic diagnoses (63/147). The positive predictive value of dysmorphic status was only 26.3%, and the negative predictive value of nondysmorphic status was 88.7%.

**Conclusions:**

Dysmorphology‐based prediction of genetic disorders is limited because of diagnoses found in apparently isolated CHD. Our findings represent one of the only assessments of phenotype‐based screening for genetic disorders in CHD and should inform clinical genetics evaluation practices for pediatric CHD.

## Graphical abstract

Overview of study design and results—Dysmorphic patients had a two‐fold increased risk of having genetic disorders identified, consistent across extra cardiac anomalies (ECA) status. However, dysmorphic status as a screen for genetic disorders is limited due to 8% of genetic disorders missed due to not having ECA or noticeable dysmorphisms.

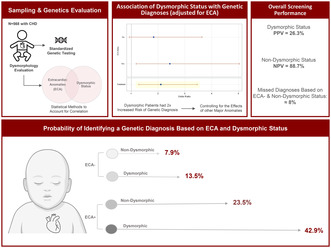

## 1 Introduction

Congenital heart disease (CHD) represents the most common type of birth defect in humans, and Mendelian genetic causes are currently identified in up to 20%–30% of patients (Cowan and Ware 2015). Syndromic CHD presentations, defined here as CHD plus additional extracardiac anomalies (ECAs) and/or dysmorphic features, account for most cases with identifiable genetic causes at present (Landstrom et al. 2021). Despite this, there are apparently isolated (nonsyndromic) CHD cases that can have Mendelian genetic causes as well, and ECA status does not sufficiently differentiate patients with underlying genetic diagnoses from those without (Helm, Landis, and Ware 2021; Shikany et al. 2020). Previous research has shown the benefits of standardizing clinical genetics evaluation and genetic testing for CHD, including improved diagnostic rates of genetic disorders and reduction in testing costs (Helm, Landis, and Ware 2021; Shikany et al. 2020; Geddes et al. 2017; Morrish et al. 2022; Blue et al. 2017). While genetic diagnoses appear to be enriched in those with syndromic presentations, research into the genetic architecture of apparently isolated CHD continues (Landstrom et al. 2021). CHD has a broad spectrum of genetic pathologies, including monogenic disorders, chromosomal aneuploidies, and chromosome copy number variation (CNV) for both syndromic and nonsyndromic CHD (Landstrom et al. 2021; De Backer and Muiño 2023). Genetic diagnosis for CHD patients is essential for informing individual/family risk stratification, genetic counseling, and patient care (Landstrom et al. 2021; Ison et al. 2022; Landis et al. 2022).

Despite the recommendations for clinical genetic evaluations and standardized diagnostic genetic testing approaches, our experience and multisite research suggest that genetic diagnoses have been underascertained for CHD (Helm, Landis, and Ware 2021; Patel et al. 2016; Hinton et al. 2015; Landis and Ware 2016). This may be due to a combination of underrecognition of genetic disorders, unfamiliarity with genetic testing advances over the last decade, and limited appreciation of the utility of a genetic diagnosis for CHD. Given the broad spectrum of genetic pathologies, CHD clinicians must develop familiarity with multiple genetic testing modalities, for example, chromosome microarray (CMA), next‐generation sequencing gene panels, and exome/genome sequencing (De Backer and Muiño 2023). Similarly, due to clinical genetics workforce limitations, many CHD patients have limited access to medical geneticists that can perform detailed dysmorphology evaluation important for syndromic/diagnostic delineation including clinical diagnoses in cases where genetic testing is negative or inconclusive (Jenkins et al. 2021; *American Journal of Medical Genetics. Part A* 2021). Despite this, previous reports have provided some insight into the importance of the noncardiac dysmorphological and ECA phenotypes in CHD (Shikany et al. 2020; Ahrens‐Nicklas et al. 2016; Goldenberg et al. 2017). However, most centers have not standardized clinical genetics and dysmorphology evaluations for CHD patients, and genetic testing remains underused (Durbin et al. 2023). Our previous work supports the benefit of implementing algorithms that standardize genetics evaluation and genetic testing for CHD to obtain early diagnoses (Helm, Landis, and Ware 2021; Shikany et al. 2020; Geddes et al. 2017).

Few studies have explored genetic diagnostic outcomes in dysmorphic versus nondysmorphic CHD in large cohorts standardizing genetics evaluations. Such studies have been difficult to conduct because they require standardized evaluations and genetic testing to minimize ascertainment bias (Shikany et al. 2020). While ECA status is the largest risk factor associated with genetic disorders, there has been less attention on the association between genetic diagnoses and minor anomalies, that is, dysmorphisms, in CHD patients (Landstrom et al. 2021). Dysmorphic features can be important but subtle physical findings that can help inform differential diagnoses and genetic testing strategies for CHD patients. Conversely, there has been relatively less attention on genetic diagnoses in the nondysmorphic CHD population, especially combined with systematic genetic testing practices for apparently isolated/nonsyndromic CHD. Our study sought to investigate genetic testing diagnostic outcomes and quantify the prevalence of genetic diagnoses identified across dysmorphic and nondysmorphic patients following standardized genetics evaluations. Our aims include the following: (1) report genetic diagnostic outcomes in CHD inpatients at our center, (2) stratify diagnoses based on ECA status and dysmorphic/nondysmorphic status and quantify the associations with diagnostic testing outcomes, and (3) assess the screening and predictive performance of dysmorphic status for genetic diagnoses confirmed by genetic testing. We show that for apparently nonsyndromic CHD, genetic diagnoses are identified but often difficult to ascertain using ECA status and dysmorphology, suggesting a role for wider population genetic testing for CHD. These findings have broader relevance for clinicians in cardiology and medical genetics who care for CHD patients and evaluate CHD populations for genetic disorders.

## 2 Methods

### 2.1 Ethical Compliance

The Indiana University Institutional Review Board deemed this study exempt after review (IRB protocol: #2004409740).

### 2.2 Overview

The study sample included primarily neonates and infants (95% ≤ age 1) with CHD referred to our inpatient cardiovascular genetics service. Our inpatient medical genetics service has standardized the evaluations of CHD patients, including assessment by board‐certified medical geneticists and genetic counselors facilitating genetic testing. We have previously discussed the clinical algorithm our center has implemented for CHD patients (Cowan and Ware 2015; Helm, Landis, and Ware 2021; Shikany et al. 2020). Generally, for the dates covered by this study, CMA testing was recommended at minimum, unless patients had previous diagnostic testing prenatally or at outside facilities prior to admission. Based on geneticist evaluations, patients may also have additional molecular genetic testing including single‐gene testing for suspected syndromes, gene panels, and/or exome sequencing/exome‐based gene panels. Generally, our program emphasized CMA and targeted molecular genetic testing (when indicated) from 2014 to 2019, and beginning in 2020, we transitioned to ES‐based testing with concurrent CNV detection/confirmation by CMA.

### 2.3 Subjects

Inclusion criteria for this study included any intensive care patient with CHD referred to our inpatient cardiovascular genetics program at Riley Hospital for Children (Indiana University Health). The representative period for this study was August 2014 through March 2021. No patients were categorically excluded if we received consults for them. We collected basic demographic data including age at consult (in days), age group (defined as neonate, infant, toddler, older child/adolescent, and adult), and race/ethnicity (self‐reported in the electronic health record system). We also collected data for the location of the admission including neonatal intensive care unit (NICU), cardiac intensive care unit (CICU), and other locations. Early in the program, cases with suspected/confirmed trisomy 21 with CHD were not routinely referred for consultation (2014–2018); starting in late 2018, these referrals routinely increased in number for diagnostic confirmation and/or additional management, but not all patients with trisomy 21 have a medical genetics consult. Internal quality improvement data from 2019 to 2021 indicated that > 97% of all inpatient CHD patients had genetic testing/evaluation.

### 2.4 Case Classification

We classified patients' CHD into mutually exclusive categories based on the system developed by Botto and colleagues for the National Birth Defects Prevention Study (Botto et al. 2007). Level 3 CHD classes included: anomalous pulmonary venous return (APVR); atrioventricular septal defects (AVSD); complex; conotruncal; heterotaxy/laterality spectrum defects; left ventricular outflow tract obstructions (LVOTO); right ventricular outflow tract obstructions (RVOTO); and septal defects. CHD was classified as “complex” for cases with multiple classes represented which could not be definitively categorized into one Level 3 class. Patients had evaluations by board‐certified medical geneticists, including documentation of ECA status, dysmorphology, clinical assessment, and recommended genetic testing strategies. For this study, ECA status was defined as the presence of any major noncardiac organ malformation(s) and/or functional anomalies with medical significance, and ECA status was dichotomized (absent/present). Examples of functional anomalies are hypotonia/hypertonia, feeding dysfunction/growth delay, and immunodeficiency. Dysmorphisms were defined as minor anatomic variations that may not have significant clinical impact but could indicate syndromic features. Dysmorphisms were defined and differentiated based on the *Elements of Morphology*, by the National Human Genome Research Institute (https://elementsofmorphology.nih.gov/). In the *Elements of Morphology* and using our classifications, dysmorphisms are represented most in the craniofacial, neck, and limb/digit regions, although we additionally include other well‐described dysmorphisms like widely spaced nipples (which is a feature not explicitly listed in *Elements of Morphology*). Dysmorphisms and ECA were extracted from the medical record at the time of the consultation and by manual review of clinical genetics documentation and review of relevant neonatal and cardiology documentation (BMH/SMW); cardiac classification ambiguities were clarified and resolved by a pediatric cardiologist experienced with embryologic classification (BJL). Data collection included variables relevant to the genetics consultation identifiable at the time of examination and within the timeframe of receiving genetic testing results. For this study, patients were categorized as “non‐dysmorphic” or “dysmorphic” based on the absence of dysmorphic features or the presence of ≥ 1 dysmorphism(s), respectively. This was based on dichotomization of the number of dysmorphisms ascertained at examination (0 vs. ≥ 1). ECA status and dysmorphology were distinguished based on guidelines for defining minor versus major anomalies in the literature and using the *Elements of Morphology* to distinguish dysmorphisms/minor anomalies (Jones and Adam 2015; Adam and Hudgins 2003). Otherwise, we also varied the thresholds of the number of dysmorphisms for defining dysmorphology status at ≥ 1 to ≥ 5 dysmorphisms and investigated associations with genetic diagnoses identified. Last, in some cases consulting geneticists documented ECAs in the dysmorphology/physical examination notes (e.g., hypotonia), although for these cases the ECA was not double‐counted (as a dysmorphic feature/ECA).

Consulting geneticists assessed each patient prior to ordering and completing genetic testing; however, exceptions to this include cases where prenatal genetic screening indicated increased risk for certain disorders (e.g., trisomy 21) based on cell‐free fetal DNA screening or other prenatal testing (needing postnatal geneticist evaluation and diagnostic confirmation). Due to the age of our patient sample, we were unable to assess for neurodevelopmental disorders or dysmorphisms/ECA that became recognizable with older age. Last, we classified each patient at the time of examination prior to genetic testing results as “apparently isolated/non‐syndromic CHD,” “apparently syndromic CHD (but not specified),” and “syndromic CHD.” The middle category was based on presence of ECA/dysmorphisms in a patient who otherwise was unable to be clinically diagnosed on examination but who was suspected of having a possible syndrome. Note that for apparently isolated/nonsyndromic descriptions, some patients could have one or few dysmorphisms or ECA present (e.g., hypotonia), but geneticists had no/low suspicion for syndromic occurrence. Otherwise, the last category was reserved for patients who had a clinically diagnosed syndrome at examination by the consultant geneticist.

### 2.5 Genetic Testing Practices

Our program had historically prioritized CMA as a first‐tier clinical genetic test for all CHD from 2014 to 2021. Beginning in 2020, our program transitioned to completing concurrent CMA with exome‐based gene panels through 2021. CMAs were performed in clinical laboratories using standard methods. In‐house CMA was performed on genomic DNA extracted from peripheral blood using the Applied Biosystems CytoScan HD array platform (ThermoFisher Scientific, Carlsbad, CA, USA) consisting of 1,953,246 unique non polymorphic copy number probes and 743,304 single nucleotide polymorphism probes spanning the whole genome. The CNVs were analyzed and reported using the NCBI human genome build 37.1 (GRCh37/hg19) by board‐certified cytogeneticists. Molecular genetic testing, including exome‐based gene panels and standard exome sequencing (singleton, duo, or trio samples as able) and phenotype‐targeted next‐generation sequencing gene panels, were performed by commercial CAP/CLIA‐approved genetic testing laboratories in the United States using standard methodologies (GeneDx Inc. Gaithersburg, MD & Prevention Genetics Inc. Marshfield, WI). From 2014 to 2019, molecular genetic testing was completed based on geneticist evaluations, but in 2020–2021, exome‐based testing with concurrent CMA was standardized for the evaluation of all CHD patients (Prevention Genetics Inc. Marshfield, WI).

Genetic testing results were classified as (1) negative, (2) variant(s) of uncertain significance (VUSs), or (3) diagnostic (i.e., pathogenic or likely pathogenic results), adhering to the American College of Medical Genetics and Genomics (ACMG)/Association for Molecular Pathology (AMP) guidelines for variant interpretation when reviewing genetic testing results (Richards et al. 2015; Riggs et al. 2020). Diagnostic results were verified by combined review by laboratory geneticists/cytogeneticists and clinical genetics teams using standard variant interpretation practices. Strict interpretation was used for all VUS results, so no candidate CNVs or molecular VUSs were classified as pathogenic/likely pathogenic results without additional supporting evidence or support from previous research (Richards et al. 2015; Riggs et al. 2020). Secondary findings are not considered outcomes in this study and did not contribute to the overall diagnostic yield of genetic testing, although there could be patients with > 1 genetic disorder/syndrome diagnosis (incidental to genetic diagnoses considered causative of CHD). There were few clinical diagnoses, and these included primary ciliary dyskinesia with inconclusive or negative genetic testing but diagnostic nasal ciliary biopsy.

### 2.6 Statistical Analyses

We reported descriptive statistics using proportions/percentages for categorical data. Numeric data are presented with mean (and standard deviation) and median, when appropriate. To investigate the association between dysmorphic/nondysmorphic status and diagnostic genetic testing outcomes, we performed chi‐squared (*χ* ^2^) tests of independence. We also quantified the magnitude of this association using odds ratios (ORs) with 95% confidence intervals (CIs). To adjust for the effects of ECA status on the dysmorphic–genetic diagnostic association, we also estimated a pooled OR using the Cochran–Mantel–Haenszel *X* ^2^ test. We checked the assumption for OR homogeneity across strata using the Breslow–Day test. Inference was based on two‐sided p‐values with Type 1 error threshold of *α* < 0.05 and additionally based on review of OR 95% CIs. Supplemental analyses included screening performance of dysmorphic status (≥ 1 dysmorphism(s)) and tetrachoric correlations between binary dysmorphology thresholds (≥ 1 to ≥ 5), that is, nondysmorphic/dysmorphic status, ECA status, and genetic diagnosis identified. Additional screening metrics of dysmorphic status included the sensitivity, specificity, Youden index (J), positive and negative predictive values (PPV and NPV, respectively), and predictive summary index (PSI); interpretation was based on guidelines in the literature (Grunau and Linn 2018; Linn and Grunau 2006). Estimation of J and PSI also allowed calculation of number needed to diagnose (NND) and number needed to predict (NNP) diagnoses based on dysmorphology evaluation. Analyses were performed in SAS version 9.4 (SAS Institute, Cary, NC, USA).

## 3 Results

### 3.1 Overview of Patient Cohort

During the study period (2014–2021), there were *n* = 568 patients with CHD ascertained and complete case status, as summarized in Table 1. Our sample was comprised mostly of neonates/infants (*n* = 543/568, 95.6%), and the median age at consultation was 3 days (interquartile range [2, 14]). Our study sample was also majority male (*n* = 325/568, 57.2%), white (*n* = 412/568, 72.5%), and non‐Hispanic. These demographic data are consistent with expected population frequencies for central metropolitan Indiana.

**TABLE 1:** Characteristics of the study cohort.

Variable	Frequency (%) or median
Sex	
Female	243/568 (42.8%)
Male	325/568 (57.2%)
Age at consult (days)	Median = 3 (IQR: [2, 14])
Age group	
Neonate (0–28 days)	473/568 (83.3%)
Infant (29 days–1 year)	70/568 (12.3%)
Toddler (1–3 years)	7/568 (1.2%)
Older child/adolescent (4–17 years)	14/568 (2.5%)
Adult (≥ 18 years)	4/568 (0.7%)
Race/ethnicity group (self‐reported)	
Black	65/568 (11. %)
Hispanic/Latino	59/568 (10.4%)
Other/not reported	33/568 (5.8%)
White	412/568 (72.5%)
Inpatient location of consultation	
CICU	307/568 (54.0%)
NICU	216/568 (38.0%)
Other	45/568 (7.9%)

Abbreviations: CICU = cardiovascular intensive care unit, NICU = neonatal intensive care unit.

### 3.2 Patient Phenotypes

Tables 2 and 3 summarize relevant clinical phenotypic data collected in this study, including cardiac classification, ECA status, dysmorphic status, and completed genetic testing, stratified by those genetic diagnoses (Table 2) identified and dysmorphic status (Table 3). The most prevalent CHD classes in our sample were LVOTO and conotruncal defects (29.4% and 28.5%, respectively). Most patients did not have ECA present at the time of examination (*n* = 370/568, 65.1%), and slightly over one‐third of patients had ≥ 1 ECA present (*n* = 198/568, 34.9%). This is consistent with the previous literature showing presence of ECA in about 20%–30% of CHD patients (Cowan and Ware 2015), although our results may be influenced by medical complexity requiring NICU/CICU admission and referral to our centers. Many cases had at least one dysmorphic feature noted by geneticists (*n* = 339/568, 59.7%), however, the median number of dysmorphisms per patient was 1 and ranged from minimum = 0 to maximum = 18 (interquartile range [0, 5]). Overall, 75.9% (*n* = 431/568) of patients were described clinically as having apparently isolated/nonsyndromic CHD, and only 10.4% (*n* = 59/568) had a clinically diagnosed syndrome at the first genetics examination. The remaining 13.8% (*n* = 78/568) of patients had apparently syndromic CHD presentations at the time of examination without a recognized genetic diagnosis (Table 2).

**TABLE 2:** Clinical presentations and proportion of genetic diagnosis identified across study participants.

Variable	Cohort frequency (%)	Proportion with genetic diagnosis identified (%)	*X* ^2^ or exact test *p* value
Sex			
Female	243/568 (42.8%)	59/243 (24.3%)	0.0386
Male	325/568 (57.2%)	56/325 (17.2%)	
Age group			
Neonate (0–28 days)	473/568 (83.3%)	93/473 (19.7%)	Exact *p* = 0.4571
Infant (29 days–1 year)	70/568 (12.3%)	15/70 (21.4%)	
Toddler (1–3 years)	7/568 (1.2%)	1/7 (14.3%)	
Older child/adolescent (4–17 years)	14/568 (2.5%)	4/14 (28.6%)	
Adult (≥ 18 years)	4/568 (0.7%)	2/4 (50.0%)	
Race/Ethnicity			
Black	65/568 (11.4%)	11/65 (16.9%)	0.7801
Hispanic/Latino	59/568 (10.4%)	10/59 (17.0%)	
Other/not reported	32/568 (5.6%)	7/32 (21.9%)	
White	412/568 (72.5%)	87/412 (21.1%)	
CHD category			
APVR	19/568 (3.4%)	1/19 (5.3%)	0.0218
AVSD	16/568 (2.8%)	7/16 (43.8%)	
Complex	71/568 (12.5%)	11/71 (15.5%)	
Conotruncal	162/568 (28.5%)	41/162 (25.3%)	
Heterotaxy/ laterality spectrum	47/568 (8.3%)	4/47 (8.5%)	
LVOTO	169/568 (29.4%)	32/167 (19.2%)	
RVOTO	46/568 (8.1%)	9/46 (19.6%)	
Septal	40/568 (7.0%)	10/40 (25.0%)	
Extracardiac anomaly status			
Negative	370/568 (65.1%)	40/370 (10.8%)	< 0.0001
Positive	198/568 (34.9%)	75/198 (37.9%)	
Dysmorphology status			
Nondysmorphic (0 dysmorphisms)	229/568 (40.3%)	26/229 (11.4%)	< 0.0001
Dysmorphic (≥ 1 dysmorphisms)	339/568 (59.7%)	89/339 (26.3%)	
Clinical description at examination			
Apparently isolated/nonsyndromic	431/568 (75.9%)	27/431 (6.3%)	< 0.0001
Possibly syndromic but specific syndrome not recognized	78/568 (13.8%)	30/78 (38.5%)	
Syndromic and diagnosis recognized at evaluation	59/568 (10.4%)	58/59 (98.3%)^a^	
Genetic testing Ordered/completed			
None^c^	24/568 (4.2%)	2/24 (8.3%)	< 0.0001^b^
Prenatal genetic testing	15/568 (2.6%)	10/15 (66.7%)	
Outside hospital genetic testing	9/568 (1.6%)	5/9 (55.6%)	
Targeted cytogenetic Testing (fluorescence in situ hybridization and karyotype)	19/568 (3.3%)	14/19 (73.7%)	
Chromosomal microarray	281/568 (49.5%)	46/281 (16.4%)	
Molecular genetic testing Only (phenotype‐specific panel/single gene)	8/568 (1.4%)	3/8 (37.5%)	
Exome sequencing/exome‐based panel plus Chromosomal microarray	212/568 (37.3%)	35/212 (16.5%)	
Genetic diagnosis Identified/confirmed			
No	453/568 (79.8%)		N/A
Yes	115/568 (20.2%)		
Genetic diagnosis types			
Cytogenetic Molecular Genetic/monogenic Cytogenetic and molecular genetic (> 1 diagnosis)	86/115 (74.8%) 26/115 (22.6%) 2/115 (1.7%)		N/A

Abbreviations: APVR = anomalous pulmonary venous return, AVSD = atrioventricular septal defects, LVOTO = left ventricular outflow tract obstruction, RVOTO = right ventricular outflow tract obstruction.

^a^ One of these cases had a clinical diagnosis but not a genetic diagnosis (primary ciliary dyskinesia); genetic testing in this case was inconclusive.

^b^ This only compares genetic testing ordered at the time of consultation (targeted cytogenetic testing, chromosomal microarray, molecular genetic testing only, and exome sequencing/exome‐based panel plus chromosomal microarray).

^c^ We do not present additional data on these cases which had no genetic testing. However, anecdotal data suggest this is a combination of some parental or family declines/refusals for genetic testing and logistical ordering errors (e.g., sample was not collected prior to discharge).

**TABLE 3:** Associations between dysmorphology status and relevant clinical variables^a^.

	Nondysmorphic (%)	Dysmorphic (%)	*X* ^2^ test *p*‐value
			Odds ratio [95% CI]
Sex			
Female	90/243 (37.0%)	153/243 (63.0%)	0.1682
Male	139/325 (42.8%)	186/325 (57.2%)	OR = 0.79 [0.56, 1.11] (Reference = Female)
Column total	229	339	
Extracardiac anomaly status			
No (*n* = 370)	178/370 (48.1%)	192/370 (51.9%)	**< 0.0001**
Yes (*n* = 198)	51/198 (25.8%)	147/198 (74.2%)	OR = 2.67 [1.83, 3.90] (Reference = No)
Column total	229	339	
CHD category			
APVR	11/19 (57.9%)	8/19 (42.1%)	**0.0099**
AVSD	5/16 (31.3%)	11/16 (68.8%)	
Complex	22/71 (31.0%)	49/71 (69.0%)	
Conotruncal	53/162 (32.7%)	109/162 (67.3%)	
Heterotaxy	24/47 (51.1%)	23/47 (48.9%)	
LVOTO	83/167 (49.7%)	84/167 (50.3%)	
RVOTO	17/46 (37.0%)	29/46 (63.0%)	
Septal	14/40 (35.0%)	26/40 (65.0%)	
Column total	229	339	
Genetic diagnosis identified			
No (*n* = 453)	203 (44.8%)	250 (55.2%)	**< 0.0001**
Yes (*n* = 115)	26 (22.6%)	89 (77.4%)	OR = 2.78 [1.73, 4.47] (Reference = No)
Column total	233	344	

*Note:* Bold values signifies the statistical *p*‐values.

Abbreviations: APVR = anomalous pulmonary venous return, AVSD = atrioventricular septal defects, LVOTO = left ventricular outflow tract obstruction, RVOTO = right ventricular outflow tract obstruction.

^a^ Unadjusted associations are quantified with ORs using nondysmorphic as the reference category.

### 3.3 Genetic Testing and Genetic Diagnosis Summary

**FIGURE 1:**
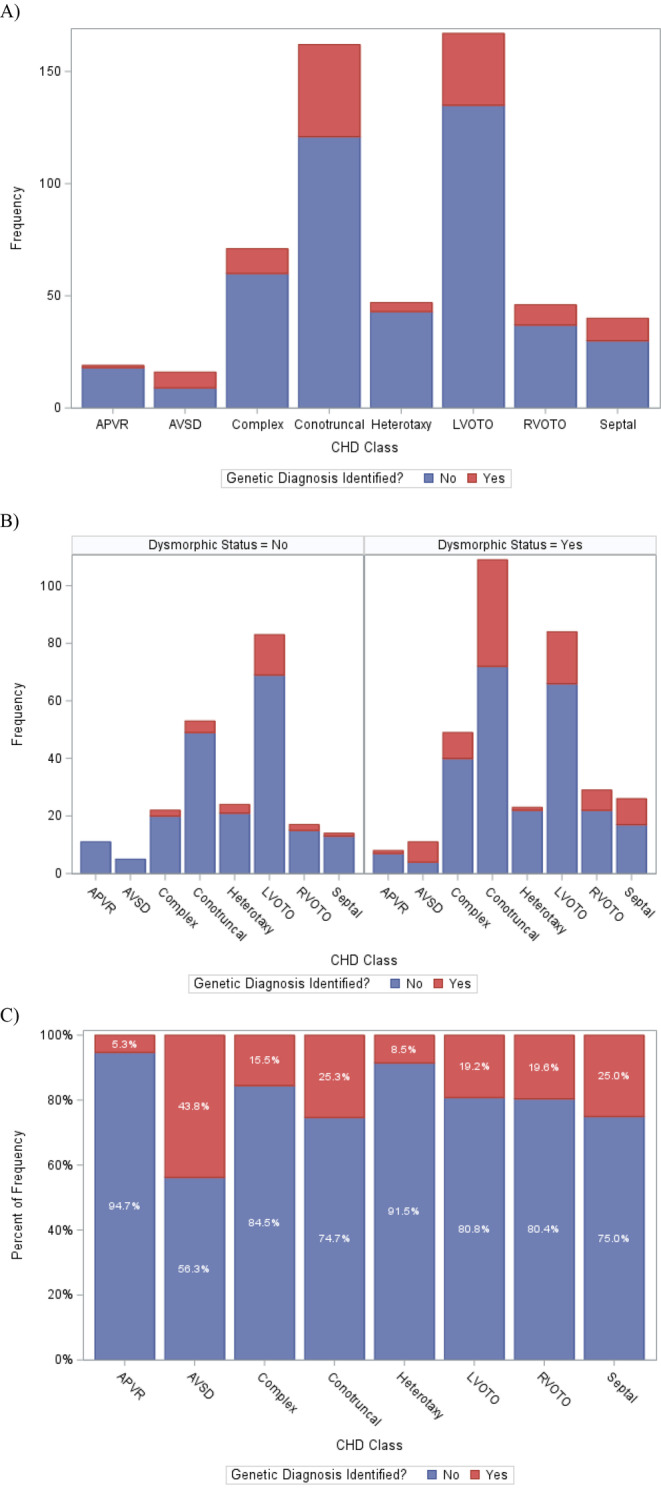
Frequency counts of genetic diagnoses identified across CHD classes (Panel A) and stratified by dysmorphology status (Panel B). Within‐class proportions of genetic diagnoses identified across CHD classes (Panel C). Proportions of genetic diagnosis within each CHD class (left panel above) are also detailed in Table 2.

The majority of patients in our study had either CMA or exome sequencing‐based testing plus CMA (86.8%, *n* = 493/568) (Table 2); the remaining patients had various genetic testing completed including molecular genetic testing (single‐gene and phenotype‐specific gene panel) and narrow cytogenetic testing such as fluorescence in situ hybridization (FISH), chromosome analysis (karyotype), prenatally completed genetic testing, or genetic testing completed at the outside hospital prior to admission (Table 2). Only 4.2% of patients (24/568) had no genetic testing completed. Overall, 20.2% of patients had an identified/confirmed genetic disorder (*n* = 115/568); details of diagnoses are summarized in Table S1. For those with diagnostic genetic testing results, 86/115 (74.8%) had a cytogenetic diagnosis, 26/115 (22.6%) had a molecular genetic diagnosis, and 2/115 (1.7%) had coinciding cytogenetic and molecular genetic diagnoses. CHD class was associated with genetic diagnosis (*p* = 0.0218); however, clinically relevant diagnoses were identified across all CHD classes. We also found that 8/568 (1.4%) of patients had multiple genetic diagnoses (i.e., ≥ 2 genetic diagnoses which could be considered incidental to the primary genetic diagnosis for CHD). The AVSD, conotruncal, LVOTO, RVOTO, and septal classes had relatively higher proportions of genetic diagnoses identified (Table 2, Figure 1). While there were more males than females in this cohort, genetic diagnoses were more prevalent in females (17.2% males vs. 24.3% females, *p* = 0.0386). ECA‐positive status and dysmorphic status were both strongly associated with genetic disorders (*p* < 0.0001). However, 10.8% of ECA‐negative patients and 11.4% of nondysmorphic patients had genetic diagnoses identified, suggesting some limitations of using ECA and dysmorphology status to screen for genetic disorders (Table 3). Table S1 summarizes narrative details of the genetic diagnoses in the patient cohort, organized by ECA and dysmorphic status.

### 3.4 Associations With Dysmorphology Status

We then investigated associations between dysmorphology status, CHD class, ECA status, and genetic diagnosis identified (Table 3). ECA‐positive and dysmorphic status were strongly associated with one another (*p* < 0.0001), with 2.7‐fold increased odds of ECA‐positive status in patients with ≥ 1 dysmorphism(s). However, over half of ECA‐negative patients had ≥ 1 dysmorphisms, showing the importance of assessing minor anomalies even in ECA‐negative patients and given the association with diagnostic genetic testing results. We also found subtle differences in dysmorphic status according to CHD class (*p* = 0.0099), with the AVSD, conotruncal, RVOTO, and septal classes having more patients with ≥ 1 dysmorphisms (Table 3, Figure 1). Overall, presence of dysmorphisms was associated with a 2.7‐fold increased risk of also having ECA present (OR = 2.67 [1.83, 3.90]). Last, genetic diagnoses were more prevalent in dysmorphic patients (*p* < 0.0001), and dysmorphic patients had a 2.8‐fold increased risk of having a genetic diagnosis identified (unadjusted OR = 2.78 [1.73, 4.47]). These results suggest that dysmorphology status is associated with ECA and genetic diagnoses which may not be easily ascertained/confirmed at the time of examination.

### 3.5 Adjusted Association Between Dysmorphology Status and Genetic Diagnoses

Next, we adjusted for the effects of ECA status on the association between dysmorphic status and genetic diagnoses (Table 4 and Figure S1). Results show that after adjusting for ECA status, there remained a strong association between dysmorphology status and genetic diagnosis identified (Cochran–Mantel–Haenszel *X* ^2^ *p* = 0.0030), with approximately twofold increased risk of genetic diagnosis being identified. Notably, for the ECA‐negative patients, there remained a positive association between dysmorphism(s) and genetic diagnoses (OR = 1.83), although it was not statistically significant within this stratum (95% CI = [0.93, 3.64]). In ECA‐negative and nondysmorphic patients, 7.9% were found to have genetic diagnoses, and this increases to 13.5% in dysmorphic ECA‐negative patients. In ECA‐positive nondysmorphic patients, 23.5% had genetic diagnoses and this nearly doubles to 42.9% in those who are ECA positive and dysmorphic (Table 5). It also suggests utility for distinguishing between ECA (major anomalies) and dysmorphisms (minor anomalies), with results showing that each is independently associated with genetic diagnosis.

**TABLE 4:** Adjusted association between dysmorphology status and genetic diagnosis identified after controlling for extracardiac anomaly status.

Extracardiac anomaly (ECA) status	Genetic diagnosis No	Genetic diagnosis Yes	Measure of association: Odds ratio [95% CI]	Common measure of association: Adjusted odds ratio [95% CI]
ECA absent				
Nondysmorphic	164/178 (92.1%)	14/178 (7.9%)	OR = 1.83 [0.93, 3.64]^a^	**OR = 2.10 [1.28, 3.46]** Cochran–Mantel–Haenszel *X* ^2^ *p* = **0.0030**
Dysmorphic	166/192 (86.5%)	26/192 (13.5%)		
ECA present				
Nondysmorphic	39/51 (76.5%)	12/51 (23.5%)	OR = **2.44 [1.19, 5.03]** ^a^	
Dysmorphic	84/147 (57.1%)	63/147 (42.9%)		

*Note:* Bold values signifies the statistical *p*‐values.

^a^ Breslow–Day test for odds ratio homogeneity (*X* ^2^ 0.3123, *p* = 0.58); this supports the validity of estimating a pooled OR.

**TABLE 5:** Proportions of genetic diagnoses stratified by extracardiac anomaly and dysmorphology status.

Extracardiac anomaly status	Dysmorphic features	Genetic diagnosis identified	Count (*n*)	Proportion genetic diagnoses, *stratum specific* (%)	Proportion genetic diagnoses, *overall cohort* (%)
No	No	No	164		
		**Yes**	14	14/178 (**7.9%**)	14/568 **(2.5%)**
	Yes	No	166		
		**Yes**	26	26/192 (**13.5%**)	26/568 **(4.6%)**
Yes	No	No	39		
		**Yes**	12	12/51 (**23.5%**)	12/568 **(2.1%)**
	Yes	No	84		
		**Yes**	63	63/147 (**42.9%**)	63/568 **(11.1%)**
Total			568		115/568 (**20.2%**)^a^

*Note:* Bold values signifies the statistical *p*‐values.

^a^ This does not include the few cases that had clinical but not genetically confirmed diagnoses.

### 3.6 Screening and Predictive Performance Metrics for Dysmorphology Status

Key screening metrics and 95% confidence intervals for dysmorphic status are summarized in Table 6. Overall sensitivity of dysmorphic status was 0.77 and specificity was 0.45, but these metrics improved depending on ECA status. For example, sensitivity increased to 0.84 in ECA‐positive patients; similarly, specificity improved to 0.50 in ECA‐negative patients. This suggests that there is overlap between ECA and dysmorphic statuses as screens for genetic disorders. The PPV of dysmorphic status was 26.3%, but the PPV ranged from 13.5% in ECA‐negative to 42.9% in ECA‐positive patients. The NPV of nondysmorphic status was 88.7% overall, but the NPV increased to 92.1% in ECA‐negative patients and decreased to 76.5% in ECA‐positive patients. Finally, as a composite assessment of predictive performance, the PSI of dysmorphic status was +14.9% (0.149). This means there is a net gain in certainty of predicting genetic diagnoses based on dysmorphic status, and PSI ranges [−1, +1], with 0 not offering any prediction and + 1 offering perfect prediction (−1 represents a screen that provides completely wrong predictions). Nonetheless, it is a relatively minimal improvement, especially compared to the prior probability of genetic disorders occurring in the population (i.e., genetic disorder prevalence in our population, at 20.2%). Some authors have suggested that PSI values of an ideal screen should be as high as 80% in the setting of population prevalence of 20% (Irving and Holden 2013), and the dysmorphic status PSI of 14.9% is considerably lower. Overreliance on ECA and dysmorphology status to inform use of genetic testing will result in missed diagnoses based on predictive values presented here, and dysmorphic classification improves prediction, but only minimally relative to population prevalence of genetic disorders.

**TABLE 6:** Performance of dysmorphology status (≥ 1 dysmorphism(s)) as screening for genetic diagnosis identified, including stratification by extracardiac anomalies status^a^.

All CHD cases		All CHD cases, stratified by ECA status	
Overall		ECA = No	ECA = Yes
Screening metrics of dysmorphology status			
Sensitivity	0.7739 [0.76975, 0.8504]	0.6500 [0.5022, 0.7978]	0.8400 [0.7570, 0.9230]
Specificity	0.4481 [0.4023, 0.4939]	0.4970 [0.4430, 0.5509]	0.3171 [0.2348, 0.3993]
Accuracy	0.5141 [0.4721, 0.5559]	0.5135 [0.4613, 0.5665]	0.5152 [0.4432, 0.5866]
Youden index	0.2220	0.1470	0.1662
Number needed to diagnose^b^	4.5	6.8	6.4
Predictive performance of dysmorphology status			
Positive predictive value (%)	26.3% [21.6%, 30.9%]	13.5% [8.7%, 18.4%]	42.9% [34.9%, 50.9%]
Negative predictive value (%)	88.7% [84.5%, 92.8%]	92.1% [88.2%, 96.1%]	76.5% [64.8%, 88.1%]
Predictive summary index (PSI)^c^ (%)	0.1490 (14.9%)	0.0568 (5.7%)	0.1933 (19.3%)
Number needed to predict^d^	6.7	17.6	5.2

^a^ The 95% confidence intervals are provided for common metrics, though these are not available for composite metrics like the Youden index and predictive summary index.

^b^ The number needed to diagnose (NND) is calculated as the inverse of the Youden index.

^c^ PSI is calculated by PPV+NPV−1, and it ranges [−1, +1], with 0 not offering any gain or loss in the predictive capability of a screen. Otherwise, PSI values > 0 indicate that a screen adds incrementally to predictive certainty above the prior probability (disease prevalence), i.e., the prevalence of genetic disorders represented in this study population.

^d^ The number needed to predict (NNP) is calculated as 1/PSI. This represents the number of patients that would need to have dysmorphology screening to correctly predict the diagnosis of one truly affected case.

### 3.7 Variable Definitions of Dysmorphic Status and Associations With Genetic Diagnoses

Last, we explored how varying definitions of “dysmorphic,” based on the number of dysmorphisms (≥ 1 to ≥ 5), alter the association with genetic diagnoses identified. We then estimated the unadjusted and ECA‐adjusted associations (Table S2). Overall, there remained a strong unadjusted association at different definitions of dysmorphic, with ORs ranging from 2.27 to 2.91. However, when adjusted for ECA status, the association remained but was attenuated at higher thresholds (ORs ranging from 1.59 to 2.10). As the threshold definition of “dysmorphic” increases from ≥ 1 to ≥ 5 dysmorphisms, the odds of diagnostic genetic testing approximately double. However, the ECA‐adjusted results suggest that in patients with higher number of dysmorphisms, a larger part of the association with genetic diagnoses may be explained by ECA status (i.e., highly dysmorphic patients were more likely to have ECA‐positive status). The descriptive relationship among dysmorphisms, genetic diagnoses identified, and ECA status is shown in Figures S2–S4. These results may help inform the future development of methods that account for the number of dysmorphisms in CHD patients as well as possible statistical interactions/correlation of dysmorphology and ECA status.

## 4 Discussion

The goal of this study was to investigate the associations between dysmorphology and genetic diagnoses in a primarily neonatal/infant population with CHD. We leveraged our clinical algorithm that standardized genetics evaluations and genetic testing for CHD patients, which has been previously described (Helm, Landis, and Ware 2021). Our results show that while ECA status is the strongest predictor of genetic diagnoses, the dysmorphology phenotype remains important for stratifying CHD patients with/without genetic diagnoses (including those without ECA). As defined in this study, the presence of ≥ 1 dysmorphism(s) is associated with approximately twofold increased risk of having a genetic diagnosis, even after adjusting for ECA status. Similarly, we found that the average number of dysmorphisms per patient was < 3, and there remained an association with genetic diagnoses at different dysmorphic thresholds (≥ 1 to ≥ 5 dysmorphisms). However, this association was attenuated at higher thresholds, likely because of correlation between highly dysmorphic patients and ECA‐positive status (with the latter accounting for most of the association with genetic diagnoses). Further investigation is needed to determine if the number of dysmorphisms would be meaningful for prediction in ECA‐negative and ECA‐positive patients. These results suggest that dysmorphology examination may provide clinical data supporting additional evaluation for ECA as part of the clinical workup (e.g., head/abdominal ultrasound), which has been reported previously; recognition of dysmorphism in patients with CHD should prompt consideration of additional ECA screening (Leppig et al. 1987; Marden, Smith, and McDonald 1964; Méhes et al. 1973). In ECA‐negative patients, 8%–14% of patients had genetic diagnoses identified, with higher prevalence in those who are dysmorphic. For ECA‐positive patients, genetic diagnoses are identified in 24%–43% of patients, with highest prevalence in dysmorphic patients. Altogether, over 20% of CHD patients in this study had a genetic diagnosis, confirming the importance of genetic evaluation in this population. Similar findings have been reported in the literature, although our study is advantaged by a larger sample size and additional standardization of genetics evaluation and genetic testing including CMA and exome‐based testing, which previous studies had not standardized (Shikany et al. 2020; Ahrens‐Nicklas et al. 2016). This study builds upon our previous work by including exome‐based testing with concurrent CMA starting in 2019–2020 (Helm, Landis, and Ware 2021).

To our knowledge, this study uniquely reports the positive and negative predictive values of ECA and dysmorphology phenotypes used to screen CHD patients for genetic disorders identified later by genetic testing. For any patient with ≥ 1 dysmorphic features, there is a 26.3% chance of diagnostic genetic testing results (PPV = 0.2625); this increases to 42.9% for ECA‐positive patients. Conversely, nondysmorphic patients have an 88.7% chance of not having a genetic diagnosis identified (NPV = 0.8865), and this increases to 92.1% in ECA‐negative patients. However, this also indicates that use of dysmorphic and ECA status could result in missed diagnoses without use of genetic testing. This ranges from 7.9% to 11.4% (using 1‐NPV), indicating a minimum prevalence of 8%–11% of CHD patients having genetic disorders despite not having overt syndromic clinical presentations. This is an important point considering the variable expression of genetic disorders as well as the difficulty in ascertaining these phenotypes in young patients. These results may support recommendations for wider implementation of genetic testing in young patients with CHD, especially if the goal is to improve earlier genetic diagnosis. Early genetics examinations would allow categorization of patients at potentially higher risk of having a genetic diagnosis prior to genetic testing results availability; conversely, nondysmorphic status in ECA‐negative patients may help reassure families and clinicians that there is lower risk for genetic disorders occurring in an infant with CHD. Given the stronger correlation between dysmorphic status and ECA‐positive status, dysmorphology evaluations can also be used to stratify risk of ECA in apparently isolated CHD and support screening.

ECA status and dysmorphology assessed by medical geneticists are imperfect proxies for identifying genetic diagnoses. However, ECA and dysmorphology are independent and additive risk factors associated with genetic diagnoses, and stratification of patients based on ECA/dysmorphology has utility for predicting diagnostic outcomes. Additionally, genetic diagnoses are identified across all CHD classes, although there is relatively higher representation among the conotruncal, LVOTO, RVOTO, and septal classes (and AVSD, influenced by trisomy 21 cases). This may emphasize a need for considering broader population genomic screening for all CHD classes since many genetic disorders are challenging to ascertain in young patients. These results will also help inform expectations for diagnostic genetic testing outcomes using the cardiac and noncardiac phenotypes at examination. The representation of genetic diagnoses among certain CHD classes was similar to findings from previous studies, with enrichment of diagnoses for the conotruncal and septal classes (Ahrens‐Nicklas et al. 2016). Ahrens‐Nicklas et al. found that 25% of their CHD sample had genetic diagnoses, similar to our findings (Ahrens‐Nicklas et al. 2016); however, the early phase of our study often did not routinely consult on cases of trisomy 21, which may have led to a relatively lower diagnostic proportion. Importantly, these investigators also confirmed the importance of considering the noncardiac phenotype (i.e., dysmorphology and ECA status) when considering genetic testing strategies. Previous studies have investigated dysmorphology in CHD patients, however, our methods were novel by quantifying the magnitude of dysmorphic versus nondysmorphic patients while also controlling for the correlation between ECA‐positive and dysmorphic status. Previous studies have instead more qualitatively classified dysmorphic/nondysmorphic status based on retrospective chart reviews by study investigators (Shikany et al. 2020; Ahrens‐Nicklas et al. 2016). Future studies may benefit from discrete quantification of dysmorphic status in addition to qualitative assessments, especially because dichotomization methods (e.g., nondysmorphic vs. dysmorphic) have been criticized for potential bias and information loss (Royston, Altman, and Sauerbrei 2006).

The results suggest that genetic diagnoses are identified in apparently isolated/nonsyndromic CHD, indicating that ECA/dysmorphology status should not stringently dictate diagnostic genetic testing strategies, especially with declining costs of such testing. Shikany et al. also found similar prevalence of diagnoses in nondysmorphic CHD patients with/without ECA, including an overall diagnostic proportion of 26% (Shikany et al. 2020). In our study, we show that both molecular and cytogenetic diagnoses are identified in the nondysmorphic subsample, suggesting a role for CMA/CNV identification and exome‐based testing for apparently isolated CHD regardless of the CHD class. The role and potential utility of broader genomic testing beyond CMA and targeted gene panels for CHD have been discussed previously (Morrish et al. 2022; Blue et al. 2022; Paige, Saha, and Priest 2018). Additional research is needed to determine the test performance, cost‐effectiveness, and clinical utility of earlier diagnoses using exome/genome sequencing with CNV detection in CHD patients in a wider range of patient populations (i.e., critical neonates/infants, children, and adults with CHD) and considering valid CHD‐associated genes (Griffin et al. 2023). Some studies suggest that for critically ill infants suspected to have genetic diagnoses, genome sequencing leads to earlier diagnoses, increases equity with access to genetic diagnosis, informs clinical management, decreases morbidity, and reduces cost of hospitalization (Krantz et al. 2021; Farnaes et al. 2018). However, broader‐scale genomic testing warrants consideration of the impacts of increasing secondary or incidental findings and a genetics healthcare workforce for handling these results. Additional investigation is needed in the CHD population, including noncritical CHD seen in older patients. Regardless, our results and previous studies show that at least 20%–25% of CHD patients have genetic diagnoses, highlighting a role for genetic evaluation in all patients with CHD in the critical care setting (Helm, Landis, and Ware 2021; Shikany et al. 2020; Ahrens‐Nicklas et al. 2016). We anticipate that earlier diagnoses in CHD infants will have clinical utility, especially considering that many of the ECA‐negative and nondysmorphic patients had genetic diagnoses associated with neurodevelopmental disorders that inform management and interventions in the first few years of life and beyond. This is particularly important, as our study shows that clinically relevant disorders with management implications are identified, including in patients with nonsyndromic presentations despite medical geneticist examinations. Such diagnoses include CNV syndromes with neurodevelopmental disorder risk like 22q11.2 deletion [OMIM #188400]/duplication [OMIM #608363] syndromes, 16p11.2 deletion syndrome [OMIM #611913], 15q11.2 deletion (BP1‐BP2) [OMIM #615656], and 8p23.1 duplication syndrome; these syndromes are challenging to identify without overt syndromic features and/or early in childhood but have longer‐term management implications (Vanlerberghe et al. 2015; Davis et al. 2019; Yu et al. 2019; Steinman et al. 2016; Barber et al. 2013). We have previously discussed cytogenetic diagnoses identified by CMA in apparently nonsyndromic CHD, including the association between 15q11.2 CNVs and CHD (Helm, Landis, and Ware 2021; Vanlerberghe et al. 2015; Davis et al. 2019).

Genetic evaluation and testing in hospitalized patients with CHD is highly institution and provider dependent (Durbin et al. 2023). This study demonstrates that genetic testing remains critical for diagnostic confirmation in apparently isolated/nonsyndromic CHD, even in settings with medical geneticist assessments as a standard for CHD evaluations. Future work is necessary to delineate genotype–phenotype correlations more clearly and develop additional methods for risk stratification and prediction. Ultimately, this work will provide data to assist clinicians' management and genetic counseling in CHD patients, prior to having results of genetic testing available. Previous work has shown that anticipating genetic diagnoses will be important, since these disorders may have different surgical management/outcomes (Landis et al. 2022). These results show the importance of the noncardiac phenotype in CHD patients, including both major and minor anomalies (ECA and dysmorphism, respectively). The presence of dysmorphic features approximately doubles the probability of diagnostic genetic testing in CHD patients, independent of ECA status. However, there was limited screening and predictive performance of ECA/dysmorphology assessment, and genetic diagnoses were found in ECA‐negative and nondysmorphic patients. The absence of ECA or dysmorphisms should not exclude consideration of genetic testing for CHD. Future work will investigate these phenotypes that may allow for improved risk stratification and prediction of genetic diagnoses in CHD patients.

### 4.1 Limitations

There are notable limitations of this study. The program's clinical algorithm prioritized CMA as a first‐tier genetic test from 2014 to 2019 and transitioned to exome‐based testing with concurrent CMA in 2020. From 2014 to 2019, decisions to proceed to molecular genetic testing following CMA were at the discretion of the consulting genetics team. Therefore, the molecular genetic diagnoses are likely underestimated in this study. CHD is broadly categorized using Botto Level 3 classifications; future studies should investigate more detailed cardiac phenotyping. Second, dysmorphology status may be impacted by the intensive care clinical environment (e.g., patient intubation, surgery, or death) resulting in incomplete ascertainment and underrepresentation. Third, these results are likely specific to the age range of the cohort and further studies will be required to determine validity for other CHD populations. Fourth, we were unable to investigate family history of CHD in this study, as these data are often incomplete or less reliably collected due to the critical care setting of the sample (and our program). This warrants future research. Last, we classified genetic diagnostic cases as those with unequivocal pathogenic/likely pathogenic findings of clinical relevance (and causal for CHD). It is possible that some of the nondiagnostic cases with inconclusive results could be reclassified in the future, resulting in an underestimate of genetic diagnoses reported here.

## Author Contributions

All authors contributed to the study conception and design. Manuscript preparation, data collection, and analysis were performed by Benjamin M. Helm and Stephanie M. Ware. Leah Wetherill contributed to statistical/analytical planning. The first draft of the manuscript was written by Benjamin M. Helm, and all remaining co‐authors commented on previous versions of the manuscript. All authors read and approved the final manuscript.

## Ethics Statement

This study was performed in line with the principles of the Declaration of Helsinki and the United States Federal Policy for the Protection of Human Subjects. Approval was granted by the Ethics Committee of Indiana University and classified as exempt (December 22, 2022, #17818). This was an observational study and consent to participate was not required. No uniquely identifying information is presented in this work.

## Conflicts of Interest

The authors declare no conflicts of interest.

## Supporting information


Table S1.

## Data Availability

The datasets generated and/or analyzed during the current study are available from the corresponding author upon reasonable request.
