# Supplementary material for: Performance of Dysmorphology‐Based Screening for Genetic Disorders in Pediatric Congenital Heart Disease Supports Wider Genetic Testing
Source: Mol Genet Genomic Med. 2024 Nov 25;12(11):e70040. doi: 10.1002/mgg3.70040 (PMC11588853; doi:10.1002/mgg3.70040)
Supplement: Supplementary file 1 — Table S1. [file MGG3-12-e70040-s001.docx]

**Online Materials – Supplemental Tables & Figures**

**Dysmorphic and Non-Dysmorphic Patients with Congenital Heart Disease: Phenotype-Based Screening Performance for Genetic Disorders**

**Journal: *Human Genetics***

Benjamin M. Helm, PhD, MS^1,2^, Lindsey R. Helvaty, BS^1^, Erin Conboy, MD^1^, Gabrielle C. Geddes, MD^1^, Brett H. Graham, MD, PhD^1^, Melissa Lah, MD^1^, Leah Wetherill, PhD^1^, Benjamin J. Landis, MD^3^, Stephanie M. Ware, MD, PhD^1,3^

Affiliations:

1. Indiana University School of Medicine, Dept. of Medical & Molecular Genetics, Indianapolis, Indiana, United States
2. Indiana University Richard M. Fairbanks School of Public Health, Dept. of Epidemiology, Indianapolis, Indiana, United States
3. Indiana University School of Medicine, Dept. of Pediatrics, Indianapolis, Indiana, United States

Corresponding Author:

Benjamin M. Helm, PhD, MS

Assistant Professor of Clinical Medical & Molecular Genetics

Indiana University School of Medicine, Dept. of Medical & Molecular Genetics

Indiana University Fairbanks School of Public Health, Dept. of Epidemiology

Email: [bmhelm@iu.edu](mailto:bmhelm@iu.edu)

ORCiD: 0000-0002-5597-0202

**Supplemental Table S1**. Summary of genetic diagnoses identified, stratified by ECA and dysmorphology phenotype.

| **Phenotype Combinations** | Non-dysmorphic + No ECA | **Diagnoses (text)** | **Diagnosis Type** | **Botto Class** | **ECA** | **Dysmorphisms** | **Case #** |
| --- | --- | --- | --- | --- | --- | --- | --- |
|  |  | 22q11.2 duplication syndrome [OMIM #608363] | Cytogenetic | Complex |  |  | 20 |
|  |  | 22q11.2 duplication syndrome [OMIM #608363] | Cytogenetic | LVOTO |  |  | 97 |
|  |  | 22q11.2 deletion syndrome [OMIM #188400] | Cytogenetic | Conotruncal |  |  | 126 |
|  |  | 16p11.2 deletion syndrome [OMIM #613444] | Cytogenetic | LVOTO |  |  | 682 |
|  |  | 16p11.2 deletion syndrome [OMIM #613444] | Cytogenetic | LVOTO |  |  | 102 |
|  |  | 15q11.2 deletion syndrome (BP1-BP2)  [OMIM #615656] | Cytogenetic | LVOTO |  |  | 230 |
|  |  | 15q11.2 deletion syndrome (BP1-BP2) [OMIM #615656] & 1p12 duplication (including portion of NOTCH2 gene) [OMIM #600275] | Cytogenetic | LVOTO |  |  | 324 |
|  |  | 8p23.1 duplication syndrome | Cytogenetic | LVOTO |  |  | 554 |
|  |  | 8p23.1 duplication syndrome | Cytogenetic | LVOTO |  |  | 152 |
|  |  | Mosaic Turner syndrome (45,X in 48/200 cells) | Cytogenetic | LVOTO |  |  | 157 |
|  |  | 17p12 deletion (including *PMP22* gene associated with HNPP) [OMIM #162500] | Cytogenetic | LVOTO |  |  | 437 |
|  |  | 11q24.2q25 deletion (Jacobsen syndrome) [OMIM #147791] | Cytogenetic | LVOTO |  |  | 548 |
|  |  | *FLT4* (c.2938del; p.Val980Serfs*27; GRCh37; NM_002020.5) (de novo)  [OMIM #136352 & OMIM #618780] | Molecular | Conotruncal |  |  | 679 |
|  |  | *NSD2* (c.3846G>A; p.Trp1282*; GRCh37; NM_007331.2) (de novo)  [OMIM #602952] | Molecular | Conotruncal |  |  | 518 |
|  | Non-dysmorphic + ECA | 22q11.2 deletion syndrome [OMIM #188400] | Cytogenetic | Conotruncal | Dysplastic kidney |  | 143 |
|  |  | Mosaic trisomy 13 | Cytogenetic | LVOTO | Myelomeningocele |  | 185 |
|  |  | 20q13 deletion syndrome | Cytogenetic | RVOTO | Hypotonia |  | 200 |
|  |  | Recombinant 8 syndrome (8.1 Mb deletion of 8p23.1p23.2 and 59.5 Mb duplication of 8q21.3-q24.3 [OMIM #179613] | Cytogenetic | Complex | Hydrocephalus, myelomeningocele |  | 226 |
|  |  | 8p23.1 duplication syndrome | Cytogenetic | LVOTO | Nevus flammeus extensive over face |  | 256 |
|  |  | 8p23.1 deletion syndrome (adult) | Cytogenetic | RVOTO | Learning disabilities |  | 428 |
|  |  | 16p11.2 deletion syndrome (w/ possible *ARMC4*-related disease with inconclusive VUS)  [OMIM #613444] | Cytogenetic | Heterotaxy | Asplenia, pancreatitis, agenesis of the dorsal pancreas, gastrointestinal malrotation |  | 618 |
|  |  | 22q11.2 duplication syndrome & *PEX1*-related peroxisomal biogenesis disorder  [OMIM #608363 & OMIM #602136] | Cytogenetic & Molecular | LVOTO | Bilateral polycystic kidney disease, hypocalcemia, intrauterine growth restriction |  | 690 |
|  |  | Diamond-Blackfan anemia (*RPL11* gene: c.396+5G>A; GRCh37; NM_000975.5)  [OMIM #612562] | Molecular | LVOTO | Hydronephrosis |  | 536 |
|  |  | Diamond-Blackfan anemia (*RPS26* gene: c.9_12delGAAA; Lys4Glufs*40) (de novo); GRCh37; NM_001029.5)  [OMIM #603701] | Molecular | Septal | Bilateral bifid thumbs |  | 675 |
|  |  | Primary ciliary dyskinesia | Clinical | Heterotaxy | Asplenia, left-sided liver |  | 328 |
|  |  | Primary ciliary dyskinesia | Clinical | Heterotaxy | Situs inversus |  | 333 |
|  | Dysmorphic  + No ECA | Trisomy 18 | Cytogenetic | Conotruncal |  | Low set and posteriorly rotated  ears with overfolded helices, wide spaced nipples, clitoromegaly, enlargement of the labia minora, wide spaced toes, increased nuchal skin | 131 |
|  |  | 22q11.2 deletion syndrome [OMIM #188400] | Cytogenetic | Septal |  | Subtle strabismus when focusing, small palpebrae, L ear with overfolded helix and low-set, cupped, prominent R ear, thin upper lip, two midline hernias (at umbilicus and one caudal to the  umbilicus), arches back when held | 145 |
|  |  | 22q11.2 deletion syndrome [OMIM #188400] | Cytogenetic | Conotruncal |  | Left ear with overfolded over superior helix, right ear crumpled | 519 |
|  |  | 22q11.2 deletion syndrome [OMIM #188400] | Cytogenetic | Conotruncal |  | Slightly overriding sutures, bulbous nose, increased nasolabial folds | 522 |
|  |  | 22q11.2 deletion syndrome [OMIM #188400] | Cytogenetic | Conotruncal |  | Square-shaped face, bulbous nose, nasolabial fold prominence, ears small and low-set | 560 |
|  |  | 22q11.2 deletion syndrome & 20q13 duplication  [OMIM #188400] | Cytogenetic | Conotruncal |  | Increased nasolabial folds and bulbous nose | 523 |
|  |  | Unbalanced 6;9 translocation (6p25.3p25.1 duplication and 9p24.2 duplication and 9q34.3 duplication due to parental balanced translocation) | Cytogenetic | Conotruncal |  | Long tapered digits, near transverse palmar crease | 176 |
|  |  | 16p11.2 duplication syndrome [OMIM #614671] | Cytogenetic | RVOTO |  | Slightly posteriorly rotated ears, hydrocele | 180 |
|  |  | 16p11.2 duplication syndrome [OMIM #614671] | Cytogenetic | LVOTO |  | Frontal bossing | 490 |
|  |  | *TBX1*-related disorder (DiGeorge syndrome) (c.1187delC; p.Pro396ArgfsX64; GRCh37; NM_005992.1)  [OMIM #602504] | Molecular | Conotruncal |  | Mild brachycephaly, mild epicanthal folds, mild underdevelopment of the superior helices, slightly posteriorly rotated ears, short nasal bridge, mild laterally displaced nipples, fair complexion | 201 |
|  |  | 15q11.2 deletion syndrome (BP1-BP2)  [OMIM #615656] | Cytogenetic | APVR |  | Mild brachycephaly, mild upslanting palpebral fissures, mild laterally displaced nipples, mild generalized hypotonia | 222 |
|  |  | 15q11.2 deletion syndrome (BP1-BP2)  [OMIM #615656] | Cytogenetic | LVOTO |  | Mild hypoplasia of the face with depression of the nasal bridge, nose is slightly slanting upward | 462 |
|  |  | Turner syndrome (45,X) | Cytogenetic | LVOTO |  | Low-set and posteriorly-rotated ears, downslanting palpebral fissures, increased neck skin folds, broad chest with widely spaced nipples | 286 |
|  |  | Turner syndrome (45,X) | Cytogenetic | LVOTO |  | Mild facial edema, fullness to the lower eyelids, low-set ears, low posterior hairline, redundant skin on neck, widely spaced nipples, excess skin over dorsal parts of the hands, sunken nails of the 1-5th toes BL | 688 |
|  |  | KBG syndrome (*ANKRD11*: c.7567C>T; p.Arg2523Trp; GRCh37; NM_013275.6)  [OMIM #148050] | Molecular | AVSD |  | Epicanthal folds, double hair whorl | 322 |
|  |  | Williams syndrome (7q11.23 deletion)  [OMIM #194050] | Cytogenetic | RVOTO |  | Stellate pattern of the irises | 475 |
|  |  | Xq28 deletion (involving *BRCC3* gene) | Cytogenetic | Conotruncal |  | Bitemporal widening of the skull, slightly low-set pinna, vertically-widened sacral dimple, very mild webbing of digits 2 & 3 on R foot | 476 |
|  |  | Costello syndrome (*HRAS*: c.34G>T; p.Gly12Cys; GRCh37; NM_005343.4)  [OMIM #218040] | Molecular | RVOTO |  | Cherubic facies, downslanting palpebral fissures, square facies, short philtrum, excess nuchal skin (excess nuchal folds) | 493 |
|  |  | *NOTCH1*-related disorder (*NOTCH1*: c.4549G>C; p.Asp1517His; GRCh37; NM_017617.5) | Molecular | Conotruncal |  | Variation of his R ear with an antihelix that extends to the tragus | 502 |
|  |  | Trisomy 21 | Cytogenetic | Septal |  | Down syndrome facies, downslanting palpebral fissures, short neck with nuchal pad, single palmar crease BL | 506 |
|  |  | Trisomy 21 | Cytogenetic | Septal |  | Prominent epicanthal folds BL, ears borderline low-set, small ears BL, short neck, nuchal fold present, significant sandal gap that is very prominent BL | 681 |
|  |  | Trisomy 21 | Cytogenetic | LVOTO |  | Telecanthus | 700 |
|  |  | Trisomy 18 | Cytogenetic | Septal |  | Short horizontal palpebral fissures, ears low set and posteriorly rotated, retro/micrognathia, redundant tissue on neck, clenched fist with overlapping digits (2 and 5 over 3 and 4 respectively | 610 |
|  |  | Ring 18 syndrome | Cytogenetic | LVOTO |  | Webbed neck, ears low-set and posteriorly rotated, neck with redundant tissue, wide-set nipples, feet narrow with positional clubbing, slightly decreased tone throughout | 629 |
|  |  | CHARGE syndrome (*CHD7*: c.2836-1G>T; GRCh37; NM_017780.4)  [OMIM #214800 & OMIM #608892] | Molecular | Conotruncal |  | Downslanted palpebral fissures, low-set ears, significant hair for age as well as a more anterior hair line, clinodactyly of the 4th and 5th toes bilaterally, the 3rd and 5th toes overlap the 4th toes  bilaterally, tapered fingers, short neck, nipples along the midclavicular line, ears with overfolding of the superior helix and a bit immature | 591 |
|  |  | *FLNA*-related disorder (*FLNA*: c.4726G>A; p.Gly1576Arg; GRCh37; NM_001456.4)  [OMIM #300017] | Molecular | LVOTO |  | Abnormal folding of the left ear | 699 |
|  | Dysmorphic + ECA | 22q11.2 deletion syndrome [OMIM #188400] | Cytogenetic | Conotruncal | IUGR, thymic aplasia, anemia, poor feeding, high-arched palate | Abnormal ears, microretrognathia, high arched palate | 2 |
|  |  | 22q11.2 deletion syndrome [OMIM #188400] | Cytogenetic | Conotruncal | IUGR, small bowel obstruction, respiratory distress, BL clubfeet | Unusual facial features, sagittal suture slightly separated, small downslanting palpebral fissures, underdeveloped nasal bridge, remarkably short sternum, BL overlapping digits, BL equinovarus, BL prominent heels | 42 |
|  |  | 22q11.2 deletion syndrome [OMIM #188400] | Cytogenetic | Complex | Cleft palate, hypospadias, hydronephrosis | L ear protuberant Bulbous nose | 5 |
|  |  | 22q11.2 deletion syndrome [OMIM #188400] | Cytogenetic | Conotruncal | Imperforate anus w/ a fistula | Mildly overfolded helices, L transverse palmar crease, hypoplastic nails of the 5th toes BL, imperforate anus noted | 105 |
|  |  | 22q11.2 deletion syndrome [OMIM #188400] | Cytogenetic | Conotruncal | Hypocalcemia, thymic hypoplasia, multicystic dysplastic R kidney | Ridging of the metopic suture, mild excessive nuchal skin, normal facial features | 158 |
|  |  | 22q11.2 deletion syndrome [OMIM #188400] | Cytogenetic | Complex | Hypotonia (mild) | Mild brachycephaly, mild reverse epicanthal folds, small moderately dysplastic L ear with loop look of the superior helix and right small ear with normal architecture; prominent nasolabial folds, mild laterally displaced nipples, mild hypotonia | 284 |
|  |  | 22q11.2 deletion syndrome [OMIM #188400] | Cytogenetic | Septal | FTT, BL choroid plexus cysts (prenatally diagnosed), hypotonia (generalized) | Mild brachycephaly, mild flat and asymmetrical occiput, slightly downslanting palpebral fissures, prominent superior helices, flat antihelices, mild down-placement of the crura and posteriorly rotated; short nasal bridge, very long and thin fingers, 2-3 overlapping toes and possible toe camptodactyly; generalized hypotonia (truncal > acral) | 319 |
|  |  | 22q11.2 deletion syndrome [OMIM #188400] | Cytogenetic | Conotruncal | IUGR, microcephaly | Subtle dysmorphism: diminished supraorbital ridges, hooded upper eyelids, small round shortened ears (2.6 cm BL which is 3rd%ile), diminished alae nasi | 340 |
|  |  | 22q11.2 deletion syndrome [OMIM #188400] | Cytogenetic | Conotruncal | Indurated area of the L neck with an overlying small skin tag (unclear etiology) | No major dysmorphisms, but he does have a ~2cm thickened somewhat indurated looking area of the L neck with an overlying small skin tag, under-riding toe on the L foot | 384 |
|  |  | 22q11.2 deletion syndrome [OMIM #188400] | Cytogenetic | Conotruncal | Cleft palate, hypocalcemia, global hypotonia | Mild brachycephaly, mild flat midface, mild left-sided facial asymmetry, right sided hemifacial microsomia, mild sunken orbits, posteriorly rotated ears, simple ears, short nasal bridge, V-shaped cleft palate, mild asymmetry of the left commissure, long fingers and digitized thumbs, mild global hypotonia | 407 |
|  |  | 22q11.21 deletion syndrome [OMIM #188400] | Cytogenetic | Conotruncal | Cleft palate | Cleft palate, downslanting palpebral fissures, ?microphthalmia, short sternum, transverse palmar creases | 421 |
|  |  | 22q11.2 deletion syndrome [OMIM #188400] | Cytogenetic | Conotruncal | Hypotonia | Hypotonic, microcephaly, prominent nasal bridge, small ears with underdeveloped creases, retrognathia, small face | 444 |
|  |  | 22q11.21 deletion syndrome [OMIM #188400] | Cytogenetic | Conotruncal | Hypotonia | Low anterior hairline, mild bitemporal narrowing, short palpebral fissures, epicanthal folds, prominent nasal root/bridge/bulbous tip, hypoplastic alae nasi, small mouth, prominent maxillary alveolar ridge, micrognathia, posteriorly rotated underdeveloped ears, mildly short neck, somewhat long fingers, minimal sacral dimple | 466 |
|  |  | 22q11.2 deletion syndrome [OMIM #188400] | Cytogenetic | Conotruncal | Hypocalcemia, feeding difficulties | Small ears, posteriorly rotated ears | 474 |
|  |  | 22q11.2 deletion syndrome [OMIM #188400] | Cytogenetic | Septal | Failure to thrive, BL vocal cord paralysis, BL tear duct stenosis, hip dysplasia, anterior anus | failure to thrive, bilateral vocal cord paralysis, mild laryngomalacia with epiglottic prolapse, normal-appearing vocal folds and arytenoids with vocal cords in a fixed midline position, minimal vocal fold excursion with respirations; bilateral tear duct stenosis, and hip dysplasia, facial asymmetry (left side of face appears smaller), anterior anus placement, and left ear placement slightly higher than right ear placement; paranasal sinus mucosal thickening and bilateral mastoid effusions | 571 |
|  |  | 22q11.2 deletion syndrome  (also with increased ~24% regions of homozygosity on CMA)  [OMIM #188400] | Cytogenetic | Conotruncal | Hypoplastic auditory canals, absent tragus | Downslanting palpebral fissures, ears with significant anomalies: left-side absent tragus and a pedunculated skin tag just medial to where tragus should be; right-side with hypoplastic tragus with similar skin tag that has a larger base compared to the left side but more widely spaced than the left; bilateral hypoplastic auditory canals | 371 |
|  |  | 22q11.2 deletion syndrome & *PAX6*-related disorder (c.279delG; p.Glu93AspfsX31; GRCh37; NM_000280.6)  [OMIM #188400 & OMIM #607108] | Cytogenetic & Molecular (> 1 Dx) | Conotruncal | Hypotonia (extremities), aniridia, ptosis bilaterally | High anterior hairline, prominent forehead, bitemporal narrowing, downslanting palpebral fissures, eyelid edema BL, small ears, low-set ears, posteriorly-rotated L ear, long toes (was later found to have aniridia as an outpatient) | 101 |
|  |  | 7q11.23 duplication syndrome  [OMIM #609757] | Cytogenetic | LVOTO | Hypospadias, hydronephrosis | Hypoplastic helices; large great toes, abdominal distension, sacral dimple | 6 |
|  |  | Seckel syndrome (*CEP152* bi-allelic mutations)  [OMIM #613529 & OMIM #613823] | Molecular | RVOTO | Microcephaly | SGA, microcephaly, sloping forehead, overriding metopic suture, beaked nose, thin lips, microretrognathia; post rotated ears; clinodactyly | 9 |
|  |  | 15q25.3q26.3 deletion of ~13.9 Mb (paternal: apparently balanced 46, XY, t(14;15)(p12;q26.1) | Cytogenetic | LVOTO | Micropenis, hydroceles, BL clubfeet (severe) | SGA 1.37 kg, Micropenis, Large ant fontanelle and post fontanelle, high forehead, dolichocephaly, downslanting palpebral fissures, periorbital edema, ears posteriorly rotated (mild), ears borderline low-set, sacral dimple, thumbs are anteriorly-displaced (mildly) | 11 |
|  |  | Alagille syndrome (*JAG1*: c.92C>T; p.Ala31Val; GRCh37; NM_000214.3)  [OMIM #601920 & OMIM #118450] | Molecular | RVOTO | Hypoplastic vessels w/ multiple collaterals, anteriorly placed anus | Anteriorly placed anus; deep sacral dimple; bulbous nose | 19 |
|  |  | Unbalanced 11;19 translocation | Cytogenetic | Septal | IUGR, hydronephrosis, iris colobomas, retinal colobomas, microphthalmia, brain anomalies | Micrognathia, flattened nasal bridge, narrow high arched palate, micro-ophthalmia, anterior-placed anus and sacral dimple | 26 |
|  |  | Noonan syndrome (*RAF1*: c.770C>T; p.Ser257Leu; GRCh37; NM_002880.4)  [OMIM #164760 & OMIM #611553] | Molecular | LVOTO | Subdural hematomas, BL cryptorchidism, micropenis | Widely split sutures, macrocephaly, tall forehead, downslanting palpebral fissures, hypertelorism, low-set ears, posteriorly-rotated ears, pointed chin, redundant neck folds/webbing, wide-set nipples, asymmetrically-placed nipples, short penis, cryptorchidism, slight hypotonia, single transverse palmar crease of the L hand, L thumb is slightly proximally placed, mild sandal gap, BL subdural hematomas | 30 |
|  |  | Turner Syndrome (45,X) | Cytogenetic | LVOTO | IUGR, short 4th metacarpals | IUGR, features c/w with Turner syndrome, pedal edema Short 4th metacarpals wide spaced nipples, sacral dimple | 44 |
|  |  | 16p11.2 deletion syndrome [OMIM #613444] | Cytogenetic | Conotruncal | Butterfly/hemi-vertebrae, rib anomalies, cystic R kidney, absent L kidney (VACTERL-like) | Upslanting palpebral fissure with flattened nasal bridge | 50 |
|  |  | 16p11.2 deletion syndrome [OMIM #613444] | Cytogenetic | Conotruncal | R hemivertebrae, rib anomalies (VACTERL-like) | R outer ear underdeveloped & posteriorly rotated; left helix slightly hypoplastic; R hemivertebrae and rib anomalies | 54 |
|  |  | 16p11.2 deletion syndrome [OMIM #613444] | Cytogenetic | LVOTO | Hypotonic | Mild clitoromegaly | 345 |
|  |  | Noonan syndrome (*RAF1*: c.770C>T; p.Ser257Leu; GRCh37; NM_002880.4)  [OMIM #164760 & OMIM #611553]) | Molecular | Conotruncal | Adducted thumbs, hypertonia | Growth parameters >95-97th%ile, LGA, trigonocephalic, broad forehead; low-set posterior-rotated ears, dysplastic cruras and small overfolded pinna; short nasal bridge, hypoplastic scrotal sac, narrow and mild hyperconvex nails, hypertonic, adducted thumbs | 136 |
|  |  | 4q26-35.2 duplication | Cytogenetic | LVOTO | Clenched fingers, hypertonia, brisk reflexes, clonus | Mild brachycephaly, short nasal bridge, low-set ears with underdeveloped superior helices, short chest with laterally displaced nipples, ruggated scrotal sac and testis not palpable, partial single palmar crease on right and left hand, clenched fingers, hypertonicity, brisk reflexes, 1-2 bits clonus (prenatal CMA identified large chromosome 4 duplication) | 149 |
|  |  | Partial trisomy 15q (~42 Mb duplication 15q11.2-q26.3) | Cytogenetic | AVSD | Absent corpus callosum, ventriculomegaly, BL polycystic kidneys | Micrognathia | 161 |
|  |  | 1q21.1 duplication syndrome  [OMIM #612475] | Cytogenetic | AVSD | Hypotonia | Mild microretrognathia, hypotonic, fetal fingertip pads present | 172 |
|  |  | Tetrasomy X | Cytogenetic | RVOTO | Genital anomalies | Hypertelorism, cranial molding, prominent occiput, overfolded ears, bruising on L side of face and head, abnormal-appearing female genitalia with L-sided enlargement of the labia and unclear anal/genital openings | 174 |
|  |  | Mowat-Wilson syndrome  (7.4 Mb deletion 2q22.1-q22.3 (encompassing *ZEB2* gene))  [OMIM #235730] | Cytogenetic | Septal | Agenesis of the corpus callosum, renal pelviectasis, hydronephrosis, abdominal distension w/ Hirschsprung's | Microcephalic by measurement, overriding sutures, prominent low-hanging columella, ears with uplifted lobes and prominent notched tragus, prominent crux of helix, pointed chin | 250 |
|  |  | Mowat-Wilson syndrome  (15.1 Mb Deletion at 2q22.1-q23.3, encompassing *ZEB2* gene)  [OMIM #235730] | Cytogenetic | Complex | Clenched fists, abdominal distension | Wide nasal bridge, short philtrum, ears with upturned/creased lobes BL, low-set ears, bridged palmar crease, clenched fists, mild-moderate abdominal distension | 352 |
|  |  | *IFT172*-Related Disorder/Ciliopathy  (c.2788-7A>G (IVS25-7A>G, homozygous; GRCh37; NM_015662.3)  [OMIM #607386] | Molecular | Heterotaxy | R atrial isomerism, midline transverse liver, R-sided spleen | Eyelid edema, downslanting palpebrae, increased hair on forehead, depressed nasal bridge, edema of face and scalp, increased nuchal skin, sacral dimple present | 359 |
|  |  | 1p36 deletion syndrome  [OMIM #607872] | Cytogenetic | Conotruncal | IUGR | Low posterior hairline, eyes slightly upslanting, eyes deep-set, mildly hypoplastic nasal root, prominent lateral nasal pillars, bulbous nose with hypoplastic alae nasi, long philtrum, mildly thin upper lip, very ridged and highly arched palate, small chin, ears mildly low set and posteriorly-rotated, thickened helices, excess nuchal skin, R single palmar crease, somewhat spatulated distal fingertips with hypoplastic nails also seen in her toes, mildly long fingers and toes | 401 |
|  |  | CHARGE syndrome  (*CHD7*: c.3226_3227delAA; p.Lys1076ValfsX9; GRCh37; NM_017780.4)  [OMIM #608892 & OMIM #214800] | Molecular | Complex | Hypertonia, cleft lip, cleft palate, hypertonic appearance | Mildly upslanting palpebral fissures, increased lanugo of forehead, mild synophrys, L cleft lip/palate, wide nasal bridge, mild flattening of the face, low-set ears, dysplastic ears BL, small chin, shortened neck, short broad chest, widely-spaced nipples, R transverse palmar crease, clenched hands BL, decreased nail width, hypoplastic toenails of feet, BL sandal gap, clinodactyly of the 4th-5th toes BL, mild 2-3 toe overlapping BL, hypertonic appearance | 194 |
|  |  | CHARGE syndrome  (*CHD7*: c.678_680delTATinsAA; p.Phe226LeufsX79; GRCh37; NM_017780.4)  [OMIM #608892 & OMIM #214800]) | Molecular | RVOTO | Cleft lip, cleft palate, DD, hearing loss, cryptorchidism, cranial nerve dysfunction | Low-set ears BL, hypoplastic antihelix and underdeveloped auricle/lobe, classic linear shape of crura, phallus appears small with prominent fat pad, undescended testes, hockey-stick palmar crease BL; | 418 |
|  |  | CHARGE syndrome  (*CHD7*: c.6070C>T; p.Arg2024Ter; GRCh37; NM_017780.4)  [OMIM #608892 & OMIM #214800]) | Molecular | Conotruncal | Seizures, optic nerve coloboma, choanal atresia | Slightly upturned nose, ears with overfolded helices BL, penis < 2 cm, nevus simplex on glabella and upper eyelids, acrocyanosis | 425 |
|  |  | Williams syndrome  (7q11.23 deletion by FISH)  [OMIM #194050] | Cytogenetic | LVOTO | Inguinal hernia | Microcephalic, broad forehead, bitemporal narrowing, periorbital fullness, epicanthal folds, pinna somewhat cupped, wide mouth, broad nasal tip, small testes, R inguinal hernia | 458 |
|  |  | Alagille syndrome: *JAG1* deletion/Alagille  7.8 Mb Deletion at 20p12.3-20p12.1 encompassing the *JAG1* gene  (there is also an AD Kallman syndrome-like gene in the affected region)  [OMIM #118450] | Cytogenetic | Conotruncal | Posterior embryotoxon | Microcephalic, slight facial asymmetry, slightly shortened R palpebral fissure, continuous horizontal palmar crease of the L hand (R hand was wrapped and unexamined); posterior embryotoxon c/w Alagille | 461 |
|  |  | Unbalanced chromosome abnormality (3;12) | Cytogenetic | Conotruncal | Micropenis, BL cryptorchidism | Microretrognathia | 486 |
|  |  | 5p13.2-5p11 duplication (10.7 Mb) | Cytogenetic | AVSD | Large and protruding tongue, umbilical hernia, hypotonia/weak suck | Mildly macrocephalic, posteriorly-rotated and low-set ears, L preauricular pit, large and slightly protruding tongue, redundant mucosal tissue on perianal skin, small umbilical hernia, small shallow sacral dimple, slight hypotonia possibly c/w sedation, weak suck | 501 |
|  |  | Noonan syndrome (*SOS1* c.1288_1293del, de novo; GRCh37; NM_005633.4)  [OMIM #182530 & OMIM #610733] | Molecular | RVOTO | Tracheobronchomalacia, IVH, feeding dysfunction, cryptorchidism, low lying conus medullaris, GER, platelet dysfunction, hypertonic in limbs (spasticity/clonus), duodenal inversus | Nuchal edema, hydrops, BL ptosis, downslanting palpebral fissures, strabismus, low-set ears, posteriorly-rotated ears, broad nasal bridge, high-arched palate, soft/doughy skin, hands with deep palmar creases | 553 |
|  |  | *PEX5*-related peroxisomal biogenesis disorder (biochemically confirmed)  (c.826C>T; p.Arg276*; paternal & c.642+1G>A; splice-site; maternal; GRCh37; NM_000319.5)  [OMIM #600414] | Molecular | Conotruncal | Hypotonia, undescended testes, BL clubfeet | Hypotonic, hypertelorism, flat and shortened nasal bridge, low set and posteriorly rotated ears, broad chest with widely spaced nipples, scrotum with little rugae present, undescended testes, BL clubfeet present, single palmar crease on L hand | 562 |
|  |  | Alagille syndrome  (*JAG1*: c.640C>T; p.Gln214*; GRCh37; NM_000214.3)  [OMIM #118450 & OMIM #601920] | Molecular | RVOTO | MAPCAs, hypocalcemia, butterfly vertebrae, hypotonia | Flat occiput, prominence of the L forehead | 627 |
|  |  | *PUF60*-related disorder/Verheij syndrome  (*PUF60*: c.658G>T; p.Glu220*; GRCh37; NM_014281.5)  [OMIM #604819 & OMIM #615583] | Molecular | Conotruncal | Ambiguous genitalia | Downslanting palpebral fissures, prominent forehead, prominent occiput, depressed nasal bridge, bulbous nose, micrognathia, thin lips, low-set ears, low hairline with hypertrichosis, small phallic structure with urethral opening, underformed scrotal tissue, general hypertrichosis, mild brachydactyly of the fingers, wide-set nipples; | 630 |
|  |  | *NOTCH1*-related disorder  (*NOTCH1*: c.5389del; p.Leu1797*; GRCh37; NM_017617.5)  [OMIM #190198] | Molecular | Complex | immunodeficiency (T-cell deficiency, macrophage activation syndrome), epilepsy, | Prominent cheeks, small forehead with slightly upslanting palpebral fissures, clubbing of fingers and toes | 631 |
|  |  | Noonan syndrome/*SOS1*-related disorder  (*SOS1*: c.508A>G; p.Lys170Glu; NM_005633.4)  [OMIM #182530 & OMIM #610733]) | Molecular | AVSD | Pleural effusions | Hypertelorism, shallow orbits, downslanting palpebral fissures, low-set and posteriorly rotated ears, dysplastic ears, broad nasal bridge, thickened nasolabial folds, low-set & wide-set nipples, apparent micromelia, hand with transverse palmar creases, fingers with broad thumbs, mild edema throughout | 636 |
|  |  | *COL11A1*-related disorder  (COL11A1: c.3816+1G>A; GRCh37; NM_001854.4)  (AKA: IVS50+1G>A)  [OMIM #120280] | Molecular | LVOTO | Mild grade 1 L hydronephrosis | Proptosis, downslanting frontal bone and forehead, large open anterior fontanelle, open posterior fontanelle, left coronal suture ridging, joined right palmar crease, large space between 1st-2nd toes BL, hypospadias | 638 |
|  |  | *ATRX*-related disorder  (*ATRX*: c.536A>G; p.Asn179Ser; GRCh37; NM_000489.6)  [OMIM #300032] | Molecular | Conotruncal | Hypotonia | Downslanting palpebral fissures, low-set and posteriorly rotated ears, dysplastic ears, hypotonia, transverse crease on left hand | 654 |
|  |  | Kabuki syndrome (*KMT2D*)  *KMT2D*: c.15079C>T; p.Arg5027*, de novo; GRCh37; NM_003482.4)  [OMIM #602113  Also another paternally inherited *RYR1* variant that is suspected to be a risk factor for malignant hyperthermia (c.4093G>T; p.Gly1365*) | Molecular | LVOTO | Hypothyroidism, R thalamic stroke | Long palpebral fissures, long eyelashes, comparatively large/prominent ears, tented lips, arched brows | 665 |
|  |  | 19p13.3 deletion syndrome/8p23.1 duplication syndrome | Cytogenetic | Complex | BL pelviectasis | Long palpebral fissures, wide nares, prominent forehead without frontal bossing, 2-3 toe syndactyly BL, prenatal CMA showed several abnormalities | 677 |
|  |  | 5q31.3-q35 duplication (~38 Mb) (non-terminal) | Cytogenetic | LVOTO | Hypoplastic R thumb with proximal insertion, bifid scrotum, anteriorly-displaced anus | Edematous appearance, eyes with small length between the inner canthi, small ears BL, R thumb with proximal insertion, bifid scrotum, grade IV hypospadias, anteriorly displaced anus | 680 |
|  |  | Partial Trisomy 5p resulting from unbalanced translocation 5;13 (causing 56.5 Mb duplication of 5p15.33-5q11.2 and a 3.46 Mb deletion of 13q11-q12.11) | Cytogenetic | Conotruncal | Left hydronephrosis, R multicystic kidney, BL clubfeet, Dandy-Walker malformation, ventriculomegaly, global hypotonia | Short narrow palpebral fissures, facial edema (microphthalmia may be present but edema made for challenging exam of the orbits), ears are small, ears are square-shaped, flat nasal bridge, micrognathia, coarse facial features, broad forehead, overlapping toes, clenched fists | 703 |
|  |  | Trisomy 13 | Cytogenetic | Conotruncal | Microphthalmia, BL postaxial polydactyly, IUGR, R-sided congenital diaphragmatic hernia | Microphthalmia, midface hypoplasia, bilateral postaxial polydactyly, IUGR, possible right-sided congenital D-hernia | 412 |
|  |  | Trisomy 13 (mosaic) | Cytogenetic | LVOTO | Postaxial polydactyly, capillary hemangiomas | Post-axial non-osseous polydactyly, capillary hemangiomas, overfolded ears, somewhat short palpebral fissures, epicanthal folds, prominent nasal root and bridge, hypoplastic alae nasi, mild micrognathia | 469 |
|  |  | Trisomy 18  (47,XX,+18) | Cytogenetic | Complex | Omphalocele, mild left cerebellar hypoplasia | Fine scalp hair, low-set ears, posteriorly rotated ears, small ears with prominent lobes, mild underdevelopment of superior helices, short sternum, overlap digits | 58 |
|  |  | Trisomy 18  (47,XX,+18) | Cytogenetic | Septal | IUGR, respiratory distress, left diaphragmatic hernia, pontocerebellar hypoplasia | Small for age, nasal canula and respiratory support made difficult dysmorphology exam; features suggestive of trisomy 18 | 395 |
|  |  | Trisomy 18 | Cytogenetic | Conotruncal | IUGR | Low-set and cupped ears, flat nasal bridge, underdeveloped supraorbital ridges, micrognathia, short sternum, overlapping fingers (2 over 3, and 5 over 4), camptodactyly of all digits | 639 |
|  |  | Trisomy 21 | Cytogenetic | Complex | Duodenal atresia | Features c/w Down syndrome, occipital flattening, upslanting palpebral fissures | 4 |
|  |  | Trisomy 21 | Cytogenetic | Complex | Hypotonia, absent mid-phalange of the L 5th finger | Dolichocephaly, flat facial profile, upslanting palpebral fissures, prominent epicanthal folds, low-set ears, posteriorly-rotated ears, short and flat nasal bridge, excess posterior nuchal fold, laterally and downwardly displaced nipples, BL single palmar creases, short 5th fingers with short mid-phalange on the R, absent mid-phalange on the L 5th digit, prominent sandal gap, prominent vertical plantar crease, slightly acrocyanotic | 95 |
|  |  | Trisomy 21 | Cytogenetic | AVSD | Hypotonia | Features c/w trisomy 21: brachycephaly, flat occiput, mild upslanting of the palpebral fissures, simple ears, posteriorly-rotated ears, epicanthal folds, short nasal bridge, tented upper lip, tongue thrusting, mild excess nuchal fold, laterally displaced nipples, mild sandal gap, palmar creases BL, BL 5th finger mid-phalange shortening BL, hypotonia | 113 |
|  |  | Trisomy 21 | Cytogenetic | Complex | Tracheal ring with tracheal tunneling | features c/w trisomy 21 | 193 |
|  |  | Trisomy 21 | Cytogenetic | AVSD | Hypotonia (mild) | Epicanthal folds, small palpebrae, prominent tongue, posterior neck redundant skin, single palmar creases, widened space between first and second toes, somewhat low tone; possible paternal family history of trisomy 21 | 387 |
|  |  | Trisomy 21 (unbalanced translocation) | Cytogenetic | Conotruncal | Renal agenesis, and diaphragmatic eventration concerning for hernia versus left lung atresia, hypotonia | renal agenesis, and diaphragmatic eventration concerning for hernia versus left lung atresia, Flat facial profile, mild bitemporal narrowing, low set anterior hairline, Horizontal palpebral fissures, ears posteriorly rotated, pointed nasal tip and flared nasal alae, helical overfolding of the pinnae, Short-appearing neck, excess posterior neck skin, Broad chest with widely spaced nipples, Shallow sacral dimple with visible base, long slender fingers and toes with flexion at the PIP joints but no contractures | 546 |

**Supplemental Table S2**. Effects of Varying Thresholds for Dysmorphic Status and Genetic Diagnostic Outcomes. Both Unadjusted and Extracardiac Anomaly-Adjusted Odds Ratios are Presented.

| **Dysmorphic Status Definition Threshold** | **Proportion with Genetic Diagnosis Identified (%)** | **Unadjusted Odds Ratio [95% CI]** | **Cochran-Mantel-Haenszel X^2^ p-value (controlling for Extracardiac Anomalies Status)** | **Adjusted Pooled Odds Ratio [95% CI], Controlling for Extracardiac Anomaly Status** | **Proportion with Extracardiac Anomalies (%)** |
| --- | --- | --- | --- | --- | --- |
| ≥1 Dysmorphisms | 89/339 (26.3%) | 2.78 [1.73, 4.47] | **0.0030** | **2.10** [1.28, 3.46] | 152/346  (43.4%) |
| ≥2 Dysmorphisms | 75/271 (27.7%) | 2.46 [1.61, 3.77] | **0.0084** | **1.83** [1.17, 2.88] | 131/278  (46.5%) |
| ≥3 Dysmorphisms | 66/227 (29.1%) | 2.44 [1.61, 3.71] | **0.0093** | **1.80** [1.16, 2.81] | 114/232  (48.9%) |
| ≥4 Dysmorphisms | 58/180 (32.2%) | 2.76 [1.81, 4.20] | **0.0045** | **1.90** [1.22, 2.99] | 101/184  (54.4%) |
| ≥5 Dysmorphisms | 46/146 (31.5%) | 2.35 [1.52, 3.63] | 0.0482 | 1.59 [0.99, 2.53] | 85/150  (56.2%) |

**Supplemental Figure S1**. Association Between Dysmorphic Status and Genetic Diagnosis Identified, Controlling for ECA status. The stratum-specific and pooled Odds Ratios are provided from the Cochran-Mantel-Haenszel test (Panel A). The distribution of the number of dysmorphisms across extracardiac anomalies status and genetic diagnosis identified (Panel B). Acronyms: N=ECA No, Y=ECA Yes.

A)


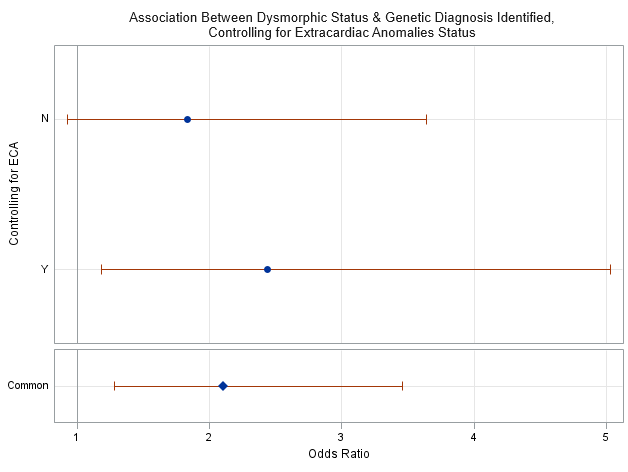


B)


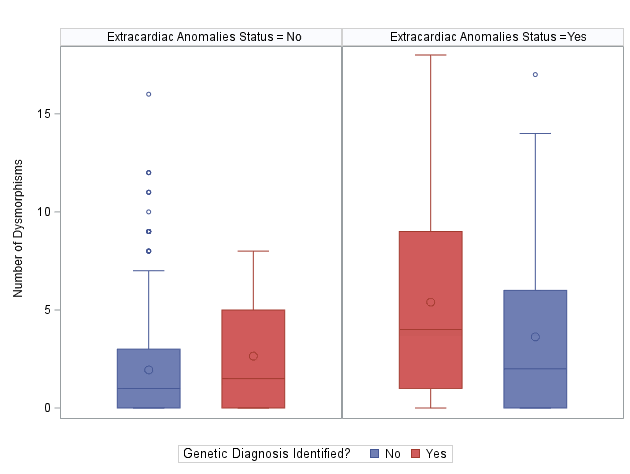


**Supplemental Figure S2**. Frequency Histogram of the Number of Dysmorphisms and Counts with Genetic Diagnosis Identified, Stratified by ECA Status.


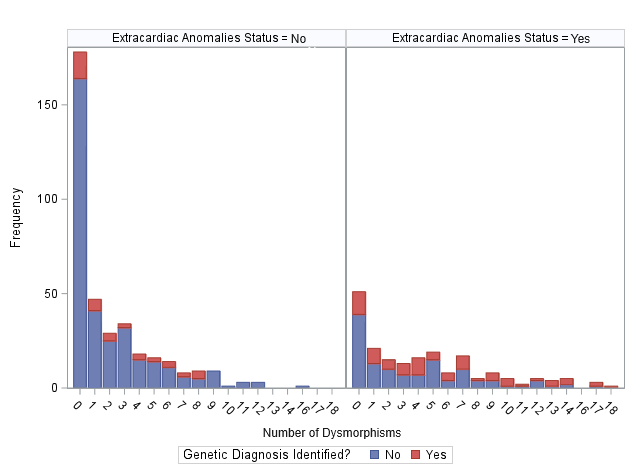


**Supplemental Figure S3**. Tetrachoric correlations indicating trends in correlation between dysmorphic thresholds and ECA (left panel)/Genetic Diagnosis Identified (right panel). At higher dysmorphic thresholds, there is an increase in correlation with ECA=Yes status; however, at higher dysmorphic thresholds, the correlation with genetic diagnosis identified decreases. Acronym: ASE=asymptotic standard error.


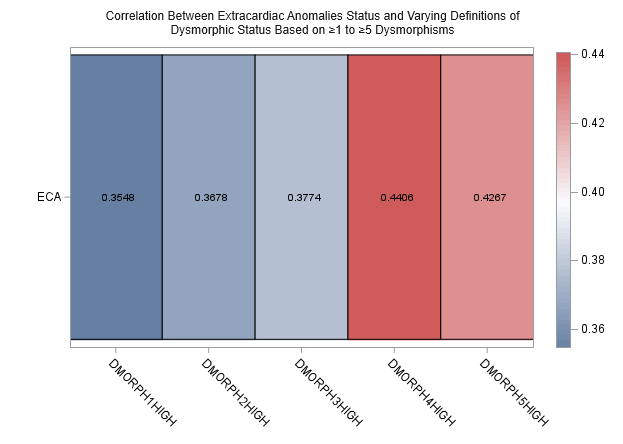

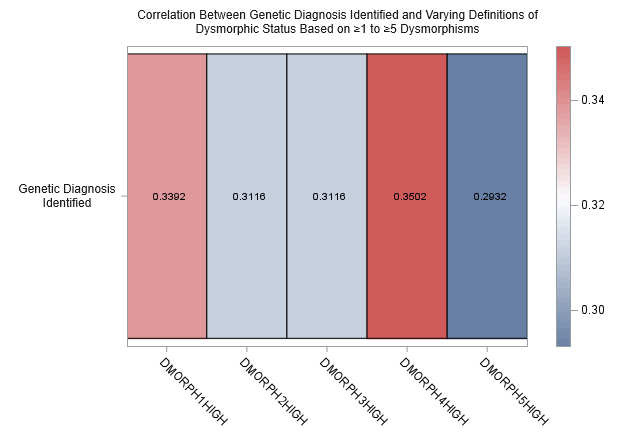


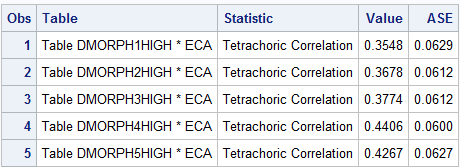

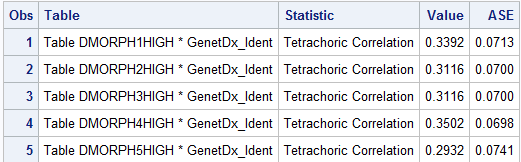


From DMORPH1 to DMORPH5, the correlation changes by +20.3% From DMORPH1 to DMORPH5, the correlation changes by -13.6%

**Supplemental Figure S4**. Tetrachoric correlations indicating trends in correlation between Dysmorphic Thresholds and Genetic Diagnosis Identified but Stratified by Extracardiac Anomalies Status. At higher dysmorphic thresholds, there is an increase in correlation with ECA=Yes status; however, at higher dysmorphic thresholds, the correlation with genetic diagnosis identified decreases.


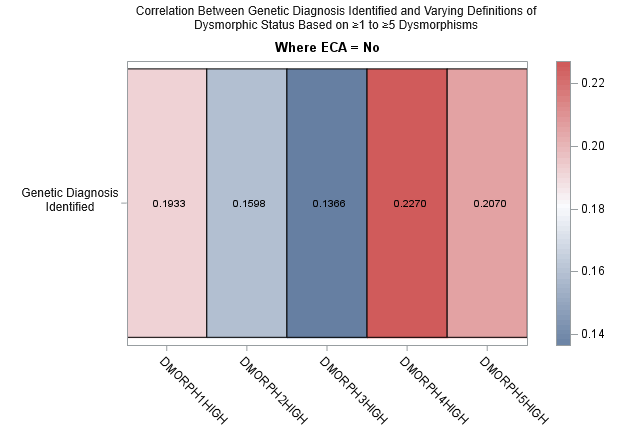

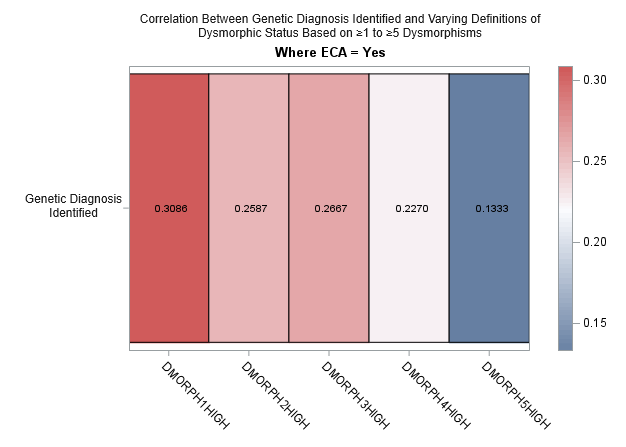


The figure above in the left shows that in ECA=No patients, the correlation between dysmorphology thresholds and genetic diagnoses identified indicates a general increase, though it is arguably minor. However, the right figure shows that in ECA=Yes patients, as the dysmorphic threshold increases, the correlation with genetic diagnosis identified decreases. Therefore, part of the association between dysmorphic status and genetic diagnosis is accounted for by the effects of ECA (i.e., at higher dysmorphic thresholds, ECA status is what is driving the association more).

This also suggests that a potential target threshold for assigning “dysmorphic” status seems to be >3 in most cases, though it may be ≥4 specifically in ECA=No patients.
